# Immune Profiling of Parkinson’s Disease Revealed Its Association With a Subset of Infiltrating Cells and Signature Genes

**DOI:** 10.3389/fnagi.2021.605970

**Published:** 2021-02-09

**Authors:** Xi Zhang, Zhihua Shao, Sutong Xu, Qiulu Liu, Chenming Liu, Yuping Luo, Lingjing Jin, Siguang Li

**Affiliations:** ^1^Stem Cell Translational Research Center, Tongji Hospital, Tongji University School of Medicine, Shanghai, China; ^2^Department of Neurology, Tongji Hospital, Tongji University School of Medicine, Shanghai, China; ^3^Key Laboratory of Spine and Spinal Cord Injury Repair and Regeneration of Ministry of Education, Orthopedic Department of Tongji Hospital, Tongji University School of Medicine, Shanghai, China

**Keywords:** Parkinson’s disease, RBM3, AGTR1, mast cell, immune cell infiltration

## Abstract

Parkinson’s disease (PD) is an age-related and second most common neurodegenerative disorder. In recent years, increasing evidence revealed that peripheral immune cells might be able to infiltrate into brain tissues, which could arouse neuroinflammation and aggravate neurodegeneration. This study aimed to illuminate the landscape of peripheral immune cells and signature genes associated with immune infiltration in PD. Several transcriptomic datasets of substantia nigra (SN) from the Gene Expression Omnibus (GEO) database were separately collected as training cohort, testing cohort, and external validation cohort. The immunoscore of each sample calculated by single-sample gene set enrichment analysis was used to reflect the peripheral immune cell infiltration and to identify the differential immune cell types between PD and healthy participants. According to receiver operating characteristic (ROC) curve analysis, the immunoscore achieved an overall accuracy of the area under the curve (AUC) = 0.883 in the testing cohort, respectively. The immunoscore displayed good performance in the external validation cohort with an AUC of 0.745. The correlation analysis and logistic regression analysis were used to analyze the correlation between immune cells and PD, and mast cell was identified most associated with the occurrence of PD. Additionally, increased mast cells were also observed in our *in vivo* PD model. Weighted gene co-expression network analysis (WGCNA) was used to selected module genes related to a mast cell. The least absolute shrinkage and selection operator (LASSO) analysis and random-forest analysis were used to analyze module genes, and two hub genes RBM3 and AGTR1 were identified as associated with mast cells in the training cohort. The expression levels of RBM3 and AGTR1 in these cohorts and PD models revealed that these hub genes were significantly downregulated in PD. Moreover, the expression trend of the aforementioned two genes differed in mast cells and dopaminergic (DA) neurons. In conclusion, this study not only exhibited a landscape of immune infiltrating patterns in PD but also identified mast cells and two hub genes associated with the occurrence of PD, which provided potential therapeutic targets for PD patients (PDs).

## Introduction

Parkinson’s disease (PD) is an age-related and the second most common neurodegenerative disorder characterized clinically by classic motor symptoms, including resting tremor, bradykinesia, rigidity, and non-motor symptoms, including insomnia, constipation, and pathologically by the progressive loss of dopaminergic (DA) neurons in the substantia nigra (SN) pars compacta and the occurrence of Lewy bodies containing α-synuclein (Cao et al., 2011; Fasano et al., 2015; Kalia and Lang, 2015). The etiology of PD has not been completely determined, involving a complex interaction between various genetic and environmental factors (Kalia and Lang, 2015). Furthermore, the pathogenesis of PD has not yet been fully understood.

Mounting evidence indicates that neuroinflammation is one of the vital features in both PD patients (PDs) and animal models of PD, the hallmarks of which are the presence of activated microglia and astrocyte that occur together with the loss of DA neurons in the midbrain (Cao et al., 2011; Calabrese et al., 2018; Stephenson et al., 2018). Microglia and astrocyte could be activated by multiple factors related to PD, such as the pivotal PD-associated genes [α-synuclein (SNCA), Parkin, deglycase (DJ-1), leucine-rich repeat kinase 2 (LRRK2)] and neurotoxins (rotenone and methyl-4-phenyl-1,2,3,6-tetrahydropyridine; Wang et al., 2015). Besides, neuroinflammation may play significant roles in pathogenetic mechanisms of PD, including the deposition of protein aggregates, injury of oxidative stress, impairment of mitochondrial function, disruption of calcium homeostasis, and abnormal iron deposition (He et al., 2020; Picca et al., 2020).

Deep researches accumulating the changes of peripheral immunity in PD patients have captured increasing attention and is gradually (Cao et al., 2011; Kannarkat et al., 2013; Lee et al., 2017). These changes are not only reflected in the types and numbers of peripheral immune cells, but also in the impact on PD-related organs, such as the intestinal tract and central nervous system (CNS; Travagli et al., 2020). Under physiological conditions, the peripheral immune cells are hardly detectable in CNS due to the existence of a blood-brain barrier (BBB). However, the pathological processes of PD could break down BBB, and BBB breakdown leads to increased infiltration of peripheral immune cells into CNS, which has been identified as one of the major contributing factors for PD (Pan and Nicolazzo, 2018; Sweeney et al., 2018). The increased infiltration of peripheral immune cells may induce excessive microglial inflammation, oxidative stress, and cytotoxicity that exacerbate neurodegeneration in PD (Wang et al., 2015; Stephenson et al., 2018; Kustrimovic et al., 2019). In a word, the current evidence indicates that there is a close relationship between the peripheral immune system and the progression of PD.

Nevertheless, the infiltration of peripheral immune cells during PD remains largely unknown, which highlights the significance to illuminate the crosstalk between the peripheral immune system and neuroinflammation in PD. Single-sample gene set enrichment analysis (ssGSEA) was developed to analyze the statistical enrichment score of each sample according to the expression of these sets of metagenes, i.e., non-overlapping sets of genes that are representative for specific immune cell subpopulations (Charoentong et al., 2017). In the present study, we used ssGSEA to calculate the immunescores of peripheral immune cells in the samples of PD patients (PDs) and healthy controls (HCs) based on their gene expression profiles available from public databases, which provided a new angle to uncover the cell types and the extent of peripheral immune cell infiltration in PD. We also identified two immune-based signature genes, RNA-Binding Motif Protein 3 (RBM3) and Angiotensin II Receptor Type 1 (AGTR1), to provide promising targets for improving the prognosis of PD patients.

## Materials and Methods

### Gene Expression Data and Processing

**Figure 1:**
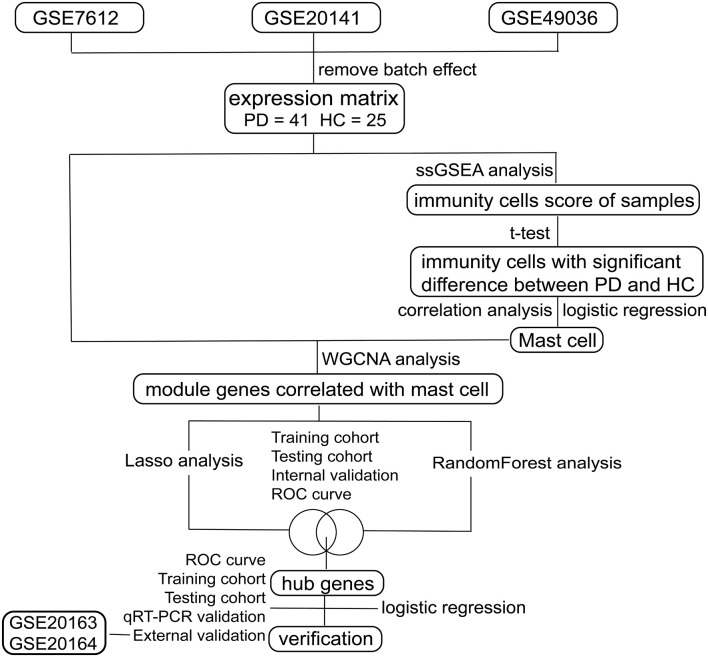
Flow chart of this study.

As shown in Figure 1, we searched the Gene Expression Omnibus (GEO^1^) for the following keywords: “(Parkinson’s disease) and (Substantia nigra)” and collected several datasets in March 2020 (Barrett et al., 2013). According to the description of these datasets, three array datasets with the same platform GPL570 were analyzed as the internal cohort in our study, including GSE7621, GSE20141, and GSE49036. The raw “CEL” files of these microarray data were downloaded, normalized, and log2 transform with “affy” packages (Gautier et al., 2004). After processing, the batch effect of three datasets was removed with “sva” packages (Leek et al., 2012), with 41 PD patients and 25 HCs subsequently included. For external validation, two array datasets with the same platform GPL96 were selected as the external cohort in our study, including GSE20163 and GSE20164. The same preprocessing procedures were performed on the two datasets as mentioned above, with 14 PD patients and 14 HCs subsequently involved; and two datasets profiled by next-generation sequencing, GSE114517, and GSE133101, were also used to verify our signature genes. The information of these datasets is listed in Table 1.

**Table 1:** The information of Gene Expression Omnibus (GEO) datasets.

GEO datasets	Platform	Method	Tissue	PD samples	HC samples
GSE7621	GPL570	Microarray	Substantia nigra	16	9
GSE20141	GPL570	Microarray	Substantia nigra	10	8
GSE49036	GPL570	Microarray	Substantia nigra	15	8
GSE20164	GPL96	Microarray	Substantia nigra	6	5
GSE20163	GPL96	Microarray	Substantia nigra	8	9
GSE114517	GPL18573	NGS	Substantia nigra	17	12
GSE133101	GPL18573	NGS	Substantia nigra	15	10

*Abbreviations: PD, Parkinson’s disease; HC, healthy control; NGS, next-generation sequencing*.

### Immune Cell Infiltration Score

The ssGSEA was introduced to quantify the relative infiltration of immune cell types in substantia nigra of each PD patients and HCs according to the expression of metagenes that are representative of specific immune cells (Jia et al., 2018); and we selected the metagene set of 28 peripheral immune cell types that were deeply researched and widely accepted, and the metagene set was shown in Supplementary Table 1 (Charoentong et al., 2017). The relative abundance of each immune cell type was represented by an enrichment score in ssGSEA analysis that was performed using “GSEA” package (Subramanian et al., 2005). Then two samples unequal variance two-tailed *t*-test was used to analyze the immunoscores of PDs and HCs to determine differential immune cell types (*p*-value < 0.05) between the two groups. We analyzed the correlations among these immune cell types and disease states and employed a Logistic regression model to evaluate the associations between immune cell types and disease occurrence using the “glmnet” package (Friedman et al., 2010). The receiver operating characteristic (ROC) curve was used to explore the sensitivity and specificity of the aforementioned model using the “ROCR” package (Sing et al., 2005).

### Weighted Gene Co-expression Network Analysis

All genes of the datasets were used for weighted gene co-expression network analysis (WGCNA) using the “WGCNA” package (Langfelder and Horvath, 2008). WGCNA was used to explore the relationships between expression modules and clinical features. The immunoscores and disease state were regarded as clinical features. According to the algorithm, we tested the independence and the average connectivity degree of different modules with different power values (the power value ranging from 1 to 20). The appropriate power value was determined when the degree of independence was 0.85 and the module construction proceeded with the appropriate power value. The minimum number of genes was set as 40 for the high reliability of the results. Module-trait associations were estimated using the correlation between module eigengenes and clinical features, which facilitates the identification of expression modules highly correlated to clinical features. Then, we selected two expression modules that had significant positive and negative correlations with clinical features and extracted the corresponding genes’ information of these modules to perform subsequent analysis.

### Identification and Validation of Signature Genes

To remove confounding genes and screen key genes without a relationship between each other, the least absolute shrinkage and selection operator (LASSO) analysis was performed to find the optimal gene list. Moreover, to select out more convincing key genes, we also chose random forest (Liaw and Wiener, 2002) for feature selection, which has been widely used and can precisely calculate the importance of each feature in the dataset. RF and LASSO analysis were performed to analyze genes’ information of two modules selected from WGCNA analysis using “randomForest” and “glmnet” packages (Liaw and Wiener, 2002; Friedman et al., 2010). Internal datasets consisting of GSE7621, GSE20141, and GSE49036 were randomly divided into the training cohort and the testing cohort by the ratio of 7-3. The intersection of two gene lists analyzed by RF and LASSO analysis in training cohort were used to construct a logistic regression model to explore the correlation of disease occurrence and these genes and were analyzed by ROC curve and confusion matrix to verify the sensitivity and specificity of the model in the testing cohort, in the internal cohort and external one.

### *In vivo* Mouse Model Experiments and Behavioral Tests

Male C57BL/6 mice (weighing 20–30 g) were purchased from Shanghai SLAC Laboratory Animal, housed, and maintained at constant temperature and humidity with a 12 h light/dark cycle in Tongji University. Eight-week-old mice (six per group) were injected a daily i.p. injection of a 1-methyl-4-phenyl-1,2,3,6-tetrahydropyridine (MPTP; Sigma–Adrich, St. Louis, MO, USA; 30 mg/kg) or saline treatment for 5 days. Motor impairments were tested with rotarod tests and pole tests. In the rotarod tests, mice were trained for 2 min at a speed of 4 r.p.m. and then performed three trials for a maximum of 4 min with increasing speed starting from 4 r.p.m. to 40 r.p.m. The pole tests were performed with a wooden pole (50 cm high, 0.5 cm in diameter, wrapped with gauze to prevent slipping) with a wooden ball at the top. After training and acclimatization, mice were tested with the pole three times for the total time it took for the mouse to get from the top to the bottom.

### Tissue Preparation

After treatment and behavioral test, mice (three per group) intended for immunofluorescence (IF) staining analysis were euthanized and transcardially perfused with PBS followed by 4% paraformaldehyde (PFA) in PBS. Brains were postfixed for 24 h in 4% PFA at 4°C and transferred to a solution of 30% sucrose in PBS for 24 h at 4°C. The coronal section of SN and STR was sectioned as 10 μm sections on a cryostat (Leica CM3050) and kept on polylysine-coated slides at −80°C. The mouse brains intended for cell lysis (three per group) were transcardially perfused with ice-cold PBS and later performed western blotting.

### Cell Co-culture and Drug Treatment

Human neuroblastoma SH-SY5Y cells and human mast cells HMC-1 (560) were kindly provided by Dr. Jingxing Zhang (Tongji Hospital, Tongji University School of Medicine, Shanghai, China) and Prof. Furong Gao (Tongji University School of Medicine, Shanghai, China), and they were cultured in Dulbecco’s Modified Eagle’s Medium (Hyclone, Logan, UT, USA) mixed with 10% fetal bovine serum (Gibco, Grand Island, NY, USA) at 37°C in a humidified incubator (Thermo Fisher Scientific, Wilmington, MA, USA) supplied with 5% CO_2_. To contribute to the two-cell co-culture system, 1 ml of 1.5 × 10^5^ cells/ml of SH-SY5Y cells and 1 ml of 1.5 × 10^5^ cells/ml of HMC-1 cells were co-cultured for 24 h directly or by using Transwell 12-well plates with 0.4 μm pore polyester membrane insert (Corning, NY, USA).

To contribute PD cell culture model *in vitro*, 1.5 ml of 1 × 10^5^ cells/ml of SH-SY5Y cells were cultured in 12-well plates for approximately 24 h and were then respectively treated with 0.1% dimethyl sulfoxide (Sigma–Adrich, St. Louis, MO, USA) containing 1 μmol/L (μM) rotenone (Sigma–Adrich, St. Louis, MO, USA) for 24 h, 1 mmol/L (mM) 1-methyl-4-phenylpyridinium (MPP^+^; Sigma–Adrich, St. Louis, MO, USA) for 24 h or 100 μmol/L (μM) 6-hydroxydopamine (6-OHDA; Sigma–Adrich, St. Louis, MO, USA) for 24 h (Feng et al., 2015; Zhang et al., 2017; Kim et al., 2018) After being digested and washed with phosphate buffer saline, SH-SY5Y cells were subjected to further treatment and analysis. Each experiment was repeated at least three times.

### RNA Extraction and Quantitative Real Time-PCR

For the quantitation of AGTR1 and RBM3 gene expression, the total SH-SY5Y cell RNA was extracted using RNAiso Plus (9109, TaKaRa, Dalian, China) following the manufacturer’s instructions. Quantitative real-time PCR was carried out using the AceQ Universal SYBR qPCR Master Mix (Q411, Vazyme, Biotech, Nanjing, China). Primer sequences are listed in Supplementary Table 2. Relative expression levels of genes were calculated by ΔΔCt method normalized to β-Actin compared with control samples.

### Western Blot Analysis

Cultured cells and brain tissues were lysed with RIPA lysis buffer (Beyotime, Wuhan, China) supplemented with protease inhibitor cocktail (Roche, Switzerland) and phosphatase inhibitor cocktail (Roche, Switzerland). BCA assay kits (Beyotime, Shanghai, China) were used to measure total protein concentrations. Then, 50–750 μg protein for each sample was separated on 12.5% SDS polyacrylamide gels with SDS running buffer [25 mM Tris (pH 8.3), 250 mM glycocoll, and 0.1% SDS]. The proteins were transferred onto 0.2 PVDF membranes (Merck Millipore, Darmstadt, Alemanha) with transfer buffer [25 mM Tris-HCl (pH 8.3), 192 mM glycocoll, and 20% methyl alcohol]. After being blocked in 5% skim milk for an hour, the membrane bars were incubated with specific primary antibodies overnight at 4°C, and then the membrane bars were washed in 0.1% TBST three times and incubated with secondary antibodies for an hour at room temperature. Antibodies are listed in Supplementary Table 3. After being washed in 0.1% TBST three times, the protein signals were detected with an ECL Western Blotting Substrate kit (Thermo Fisher Scientific, Wilmington, MA, USA) on the ImageQuant LAS 4000mini system. ImageJ software was applied for quantitative analysis of band density.

### Immunofluorescence Staining

Cultured cells on coverslips and brain slices were fixed with 4% paraformaldehyde in PBS for 15 min, washed with PBS three times, and then permeabilized in 0.1–0.3% Triton X-100 for 30–60 min. After blocking in 3% donkey serum for an hour, cells and tissues were incubated with primary antibodies overnight at 4°C. Then, cells and tissues were incubated with corresponding secondary antibodies for an hour at room temperature. Antibodies are listed in Supplementary Table 3. The nuclei were stained by DAPI (1:1,000; Roche, Switzerland) for 15 min at room temperature. Images were captured by fluorescence microscopy (OLYMPUS BX53).

### Statistical Analysis

All data are presented as mean ± standard deviation (SD). Each experiment was replicated at least three times. Data visualization and analysis were performed with GraphPad Prism 8 (GraphPad Software Inc., La Jolla, CA, USA). Statistical analysis was performed using either student’s *t*-test or one-way ANOVA. Significant difference among groups was assessed as ns *p* > 0.05, *p < 0.05, **p < 0.01, and ***p < 0.001.

## Results

### Immune Cell Infiltration Landscape of Substantia Nigra Tissue in PDs and HCs

**Figure 2:**
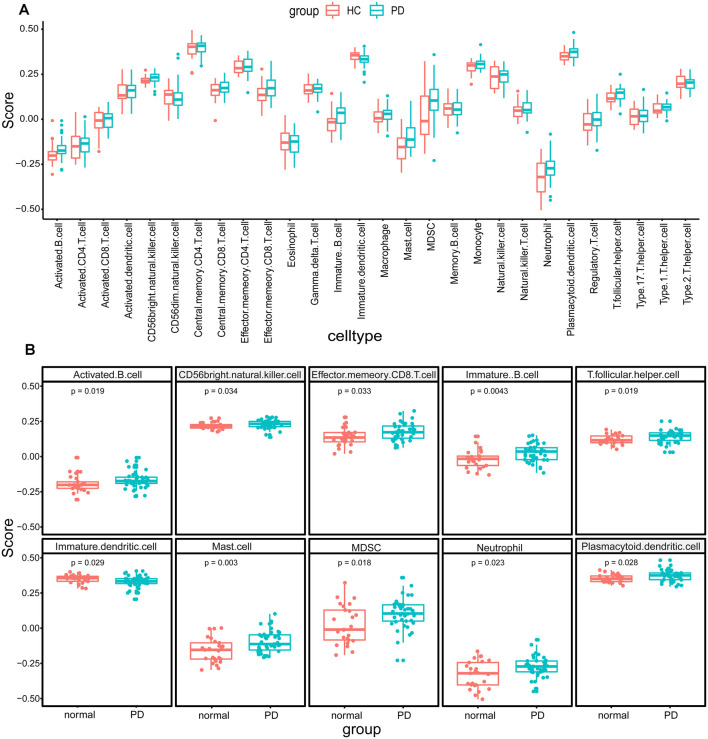
The immune cell infiltration analysis of substantia nigra between Parkinson’s disease patients (PDs) and healthy controls (HCs). **(A)** The landscape of immune cell infiltration based on expression data from the Gene Expression Omnibus (GEO) database. **(B)** The immune cell types with significant differences between PDs and HCs.

The research flowchart is shown in Figure 1. Based on the profiling data of 25 HCs and 41 PDs, the immune cell infiltration landscape of substantia nigra was constructed through ssGSEA with 28 immune cell types identified (Figure 2A). Notably, the infiltrating levels of 10 kinds of immune cells were significantly different between PDs and HCs, containing activated B cell, CD56 bright natural killer cell, effector memory CD8 T cell, immature B cell, T follicular helper cell, immature dendritic cell, mast cell, myeloid-derived suppressor cell (MDSC), neutrophil and plasmacytoid dendritic cell (Figure 2B). Together, these results suggest that the immune infiltration patterns in SN tissue of PDs might be altered during the progression of the disease.

### Mast Cell Could Be the Key Immunocyte Associated With the Occurrence of PD

**Figure 3:**
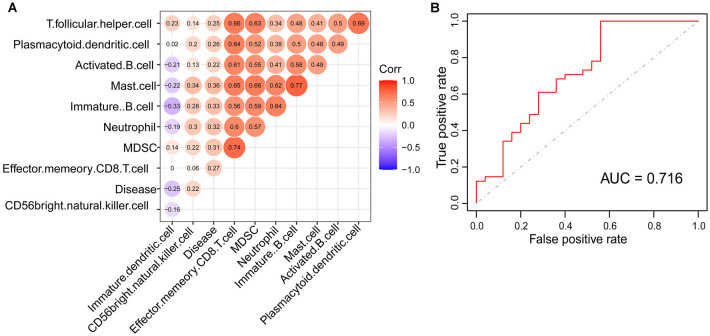
Mast cell was identified to be tightly correlated with PD. **(A)** The correlation of disease state and immune cell types with significant differences between PDs and HCs. **(B)** The receiver operating characteristic (ROC) curve of logistic model for verifying the association between mast cell and the occurrence of PD.

**Figure 4:**
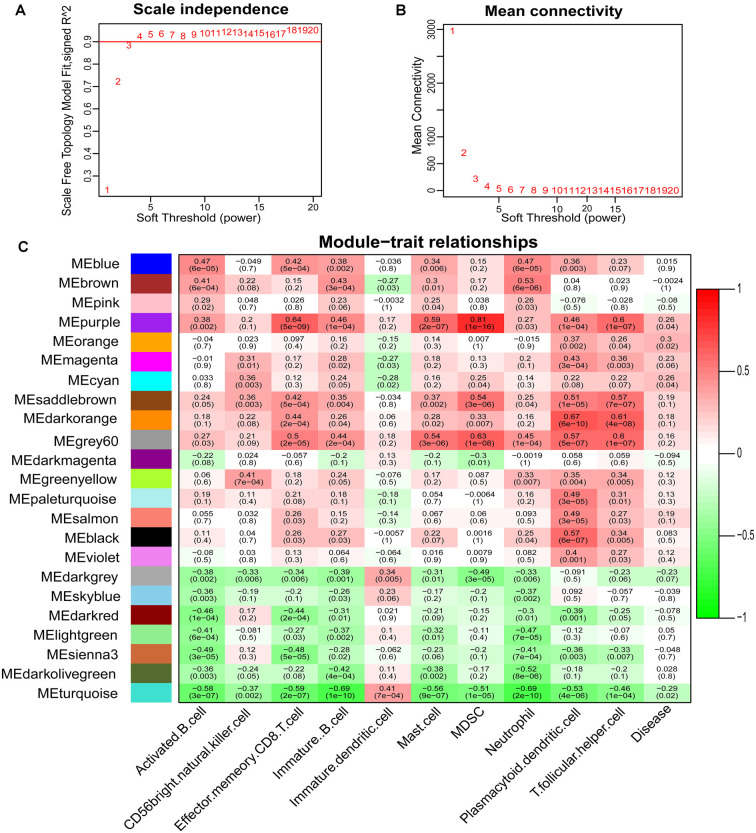
Determination of soft-thresholding power in the weighted gene co-expression network analysis (WGCNA) and identification of modules. **(A)** Analysis of the scale-free fit index for various soft-thresholding powers. **(B)** Analysis of the mean connectivity for various soft-thresholding powers. **(C)** Heatmap of the correlation between module eigengenes and clinical traits of PD.

Based on the immunoscore, we obtained immune cell types that differed between PDs and HCs. According to the correlation analysis of these immune cells and disease states, mast cells were most associated with the occurrence of PD (Figure 3A). The Logistic regression analysis based on these immune cell types revealed that mast cells were significantly correlated to the occurrence of PD, and the ROC curve of mast cells was drawn to assess the predictive accuracy with the area under the curve (AUC) = 0.716 (Figure 3B); and it was already reported that the number and activation of mast cells in PD brain slices had a higher level compared to non-PD control brain slices (Kempuraj et al., 2019). Then based on the WGCNA that was used to explore the relationships between gene expression modules and clinical features, we found that the modules significantly correlated to mast cells and disease states were the same (Figure 4). The purple module was positively correlated to a disease state (*r* = 0.26, *p* = 0.04) and mast cell (*r* = 0.59, *p* = 2e-07), and the turquoise module was observably negatively correlated to a disease state (*r* = 0.29, *p* = 0.02) and mast cells (*r* = 0.41, *p* = 7e-04). In aggregate, these results indicate that mast cells could be the key immunocyte and were most associated with the occurrence of PD.

### Multiple Algorithms Collectively Revealed That RBM3 and AGTR1 Were Associated With Mast Cell and PD

**Figure 5:**
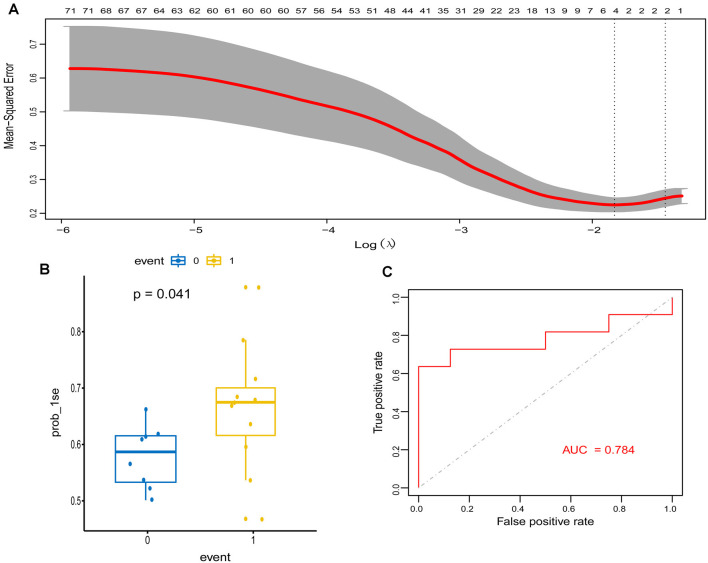
Construction and validation of the least absolute shrinkage and selection operator (LASSO) model in PD patients. **(A)** The relationship between cross-validated mean square error and model size. Partial likelihood deviance is plotted against log (*λ*), where *λ* is the tuning parameter. Dotted vertical lines were drawn at the optimal values by minimum criteria and 1-s.e. criteria, and we selected 1-s.e. criteria to construct the model. **(B)** The distribution of the LASSO model in the testing cohort. **(C)** ROC curve of LASSO model for differentiating PDs from HCs of the testing cohort.

**Figure 6:**
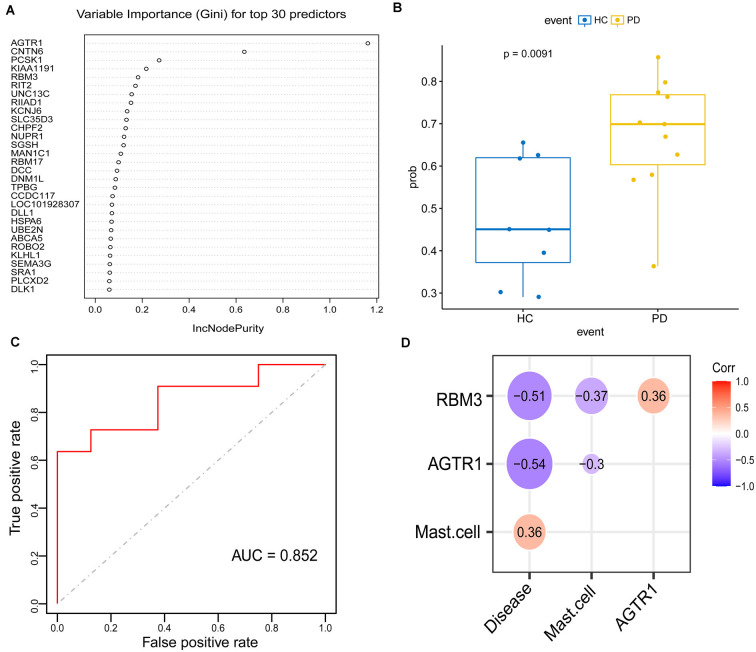
Construction and validation of the random forest (RF) model in PDs. **(A)** Top 30 genes based on the parameter of increase in node purity in RF analysis. **(B)** The distribution of the RF model is based on these 30 genes in the testing cohort. **(C)** ROC curve of RF model for differentiating PDs from HCs of the testing cohort. **(D)** The correlation between the intersection gene of lasso and RF analysis (RBM3, AGTR1), mast cell, and disease state.

To explore signature genes associated with the immune infiltration and occurrence of PD, the genes’ information of the above two modules were used to explore key genes through LASSO and RF analyses. Eight genes were identified as key genes based on LASSO analysis (Table 2, Figure 5A), and PDs and HCs could be distinguished according to the model constructed by these genes in the testing cohort (Figures 5B,C). We selected the top 30 candidate genes from the result of RF analysis (Table 2, Figure 6A), and the RF model based on these genes could also distinguish PDs from HCs in the testing cohort (Figures 6B,C). The intersection genes of the two results gained from LASSO and RF analyses, RBM3 and AGTR1, were regarded as key genes that were associated with mast cells and PD. Then the correlation analysis also indicated that RBM3 and AGTR1 were related to disease states and mast cells (Figure 6D). Collectively, these data revealed that RBM3 and AGTR1 were associated with mast cells and disease states.

**Table 2:** The key gene list of LASSO and RF analysis.

Analysis	Genes
LASSO	**RBM3**, **AGTR1**, PSPH, PAK6, MTMR9, KANK4, CALN1, TCERG1L
RF	**AGTR1**, CNTN6, PCSK1, KIAA1191, **RBM3**, RIT2, UNC13C, RIIAD1, KCNJ6, SLC35D3, CHPF2, NUPR1, SGSH, MAN1C1, RBM17, DCC, DNM1L, TPBG, CCDC117, LOC101928307, DLL1, HSPA6, UBE2N, ABCA5, ROBO2, KLHL1, SEMA3G, SRA1, PLCXD2, DLK1

*Abbreviations: LASSO, least absolute shrinkage and selection operator analysis; RF, random forest analysis. These genes in LASSO analysis are equally important. These genes in RF analysis are sorted by the parameter of increase in node purity. The two genes highlighted in bold are the overlap of the results of RF and Lasso analysis*.

### Logistic Regression Model Identified That RBM3, AGTR1 Could be Signature Genes Related to the Occurrence of PD

**Figure 7:**
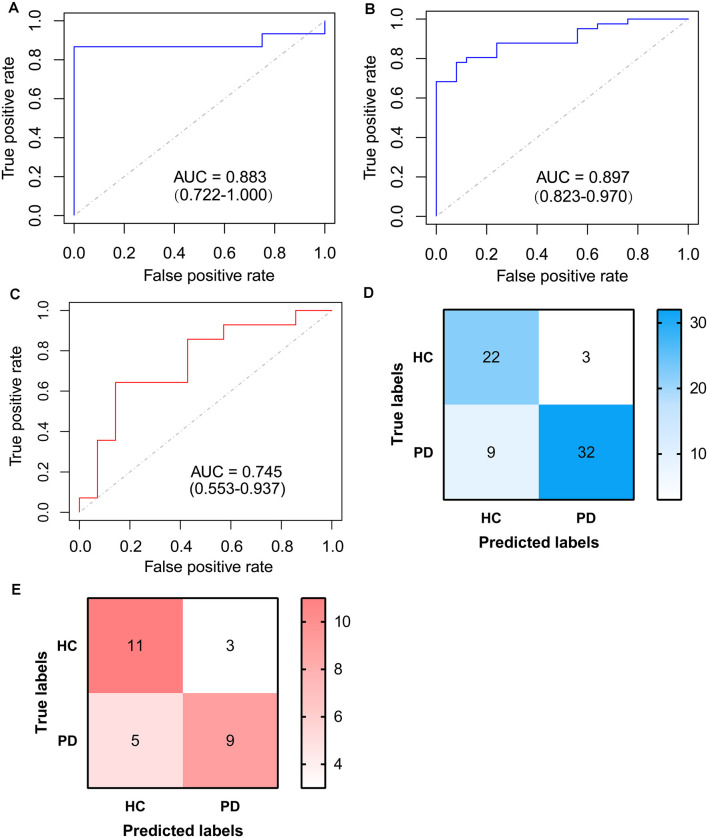
ROC curve of logistic model based on RBM3 and AGTR1. **(A)** ROC curve of logistic model for differentiating PDs from HCs of the testing cohort. **(B)** ROC curve of logistic model for differentiating PDs from HCs of the internal cohort. **(C)** ROC curve of logistic model for differentiating PDs from HCs of the external cohort. **(D)** Confusion matrix based on the internal cohort. **(E)** Confusion matrix based on the external cohort.

To identify signature genes related to the occurrence of PD, a Logistic regression model was constructed based on the expression level of RBM3 and AGTR1 in the training cohort. A ROC curve was used to evaluate the efficacy of the model in the testing cohort, the internal cohort, and the external one. The AUC of the testing cohort was 0.883 (95% CI: 0.722–1.000; Figure 7A). Moreover, the AUC of the internal cohort was 0.897 (95% CI: 0.823–0.970; Figure 7B), and the AUC of the external cohort was 0.745 (95% CI: 0.553–0.937; Figure 7C). A confusion matrix was also used to evaluate the model in the internal cohort (Figure 7D, Table 3) and the external cohort (Figure 7E, Table 3). Together, these results indicate that RBM3 and AGTR1 could be signature genes of PD.

**Table 3:** The model index of confusion matrix based on the internal cohort and the external cohort.

Index	Cohorts	
	The internal cohort	The external cohort
Accuracy	0.8182	0.7143
Precision	0.9143	0.7500
Sensitivity	0.7805	0.6429
Specificity	0.8800	0.7857

### RBM3 and AGTR1 Were Differentially Expressed Genes Between PDs and HCs

**Figure 8:**
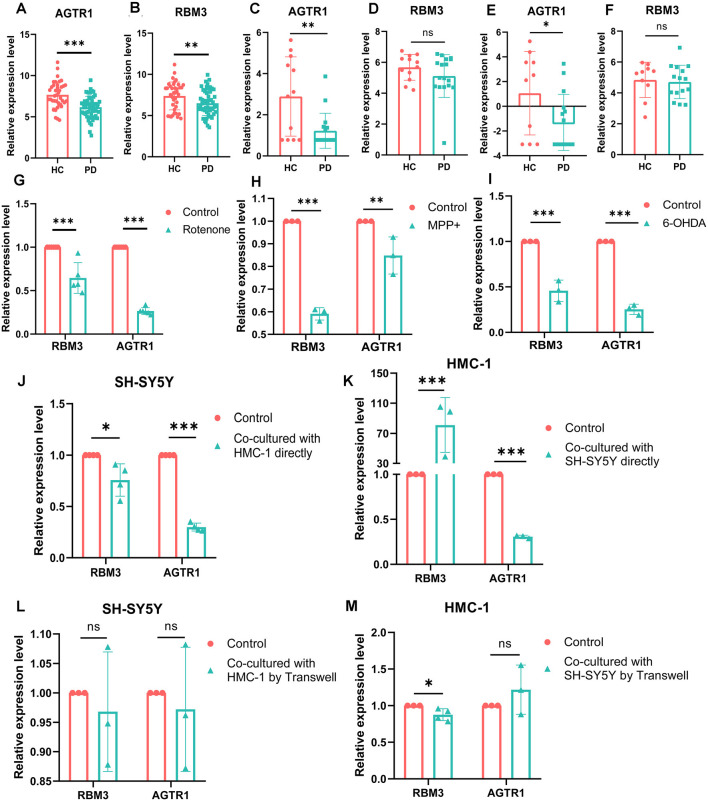
The mRNA relative expression levels of RBM3 and AGTR1 in GEO datasets and PD cell culture model. **(A)** The expression levels of AGTR1 of PDs and HCs in a total of five array datasets. **(B)** The expression levels of RBM3 of PDs and HCs in a total of five array datasets. **(C)** The expression levels of AGTR1 of PDs and HCs in GSE114517. **(D)** The expression levels of RBM3 of PDs and HCs in GSE114517. **(E)** The expression levels of AGTR1 of PDs and HCs in GSE133101. **(F)** The expression levels of RBM3 of PDs and HCs in GSE133101. **(G)** The expression levels of RBM3 and AGTR1 in cell culture model *in vitro* constructed by SH-SY5Y cell using rotenone. **(H)** The expression levels of RBM3 and AGTR1 in cell culture model *in vitro* constructed by SH-SY5Y cell using MPP^+^. **(I)** The expression levels of RBM3 and AGTR1 in cell culture model *in vitro* constructed by SH-SY5Y cell using 6-OHDA. **(J)** The expression levels of RBM3 and AGTR1 in SH-SY5Y cell co-cultured with HMC-1 cell directly. **(K)** The expression levels of RBM3 and AGTR1 in HMC-1 cell co-cultured with SH-SY5Y cell directly. **(L)** The expression levels of RBM3 and AGTR1 in SH-SY5Y cell co-cultured with HMC-1 cell by Transwell. **(M)** The expression levels of RBM3 and AGTR1 in HMC-1 cell co-cultured with SH-SY5Y cell by Transwell (ns *p* > 0.05, *p < 0.05, **p < 0.01, ***p < 0.001 vs. Control group).

To better verify the aforementioned results, we compared the expression levels of RBM3 and AGTR1 in all the array datasets, including 55 PDs and 39 HCs, and the expression levels of RBM3 and AGTR1 in PDs were significantly downregulated compared with HCs (Figures 8A,B). The relative expression levels of AGTR1 in next-generation sequencing data (Figures 8C,E) also had a significant difference between PDs and HCs, but the relative expression levels of RBM3 in PDs only shown the lower level without significant differences (Figures 8D,F). In a word, low expression of AGTR1 and RBM3 in PD patients was not only found in the microarray profiling cohort, but also in the next-generation sequencing cohort.

### RBM3 and AGTR1 Were Also Differentially Expressed Genes in PD Models

**Figure 9:**
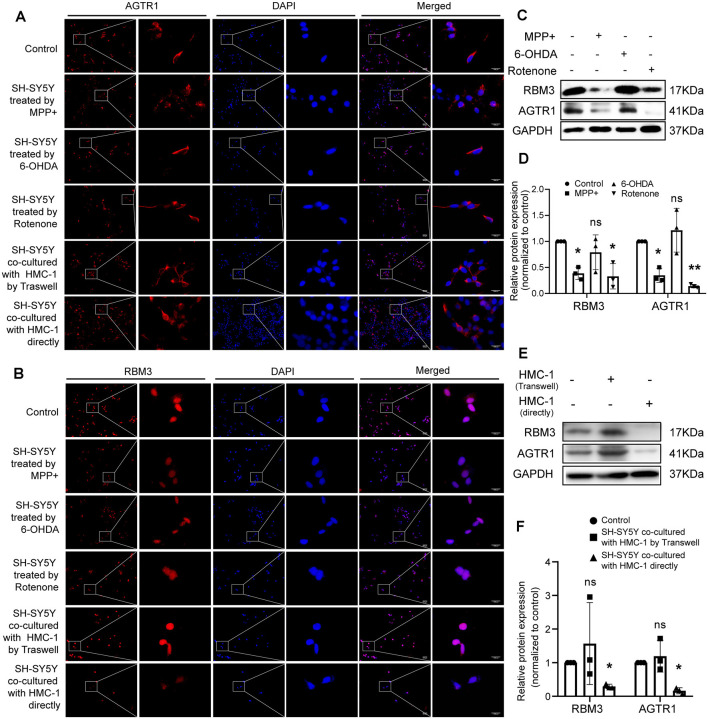
The protein relative expression levels of RBM3 and AGTR1 in PD cell culture model. **(A)** AGTR1 expression level in each treated group was detected by fluorescence microscope after immunofluorescence staining. Scale bars: 100 μm; 1,000 μm. **(B)** RBM3 expression level in each treated group was detected by fluorescence microscope after immunofluorescence staining. **(C,D)** Western blot analyses of specific genes’ expression in SH-SY5Y cells treated by MPP+, 6-OHDA, and Rotenone. GAPDH was used as an endogenous control. **(E,F)** Western blot analyses of specific genes’ expression in SH-SY5Y cells co-cultured with HMC-1 by Transwell or directly. GAPDH was used as an endogenous control (ns *p* > 0.05, *p < 0.05, **p < 0.01, vs. Control group).

**Figure 10:**
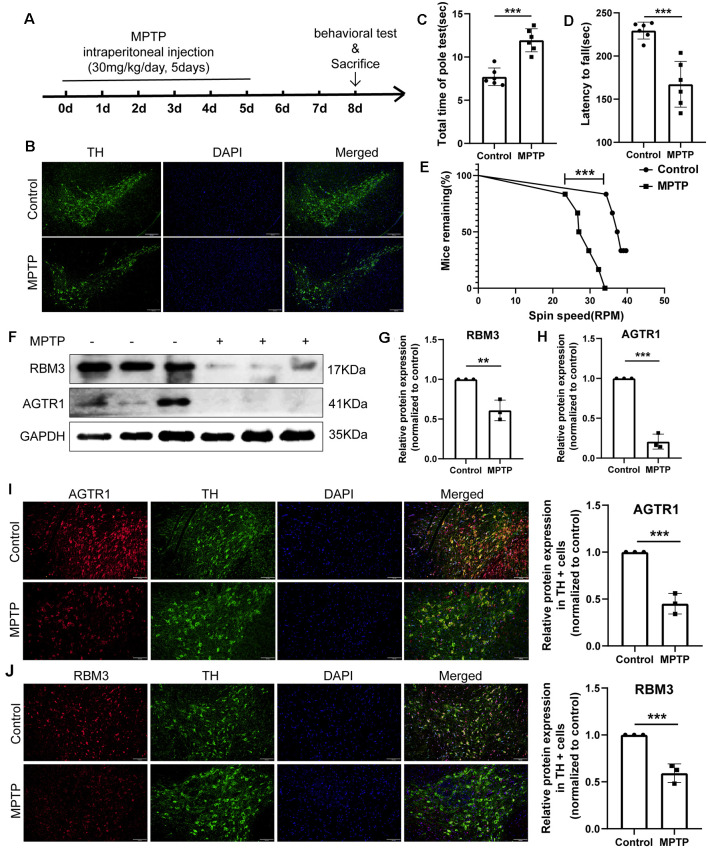
The relative protein expression levels of RBM3 and AGTR1 in the PD animal model. **(A)** The flowchart of the construction of the MPTP subacute model, behavioral tests, and sacrifice. **(B)** Tyrosine hydroxylase (TH) staining of the substantia nigra (SN) of the above mice. Scale bars: 200 μm. **(C)** Pole tests were conducted by a blinded observer after MPTP treatment. **(D,E)** Rotarod tests were conducted by a blinded observer after MPTP treatment. **(F–H)** Western blot analyses of RBM3 and AGTR1 in SN of the above mice. **(I)** The co-localization of AGTR1 and TH in SN of the control group and MPTP group was detected by fluorescence microscope after immunofluorescence staining. Scale bars: 100 μm. **(J)** The co-localization of RBM3 and TH in SN of two groups was detected by fluorescence microscope (**p < 0.01, ***p < 0.001 vs. Control group).

**Figure 11:**
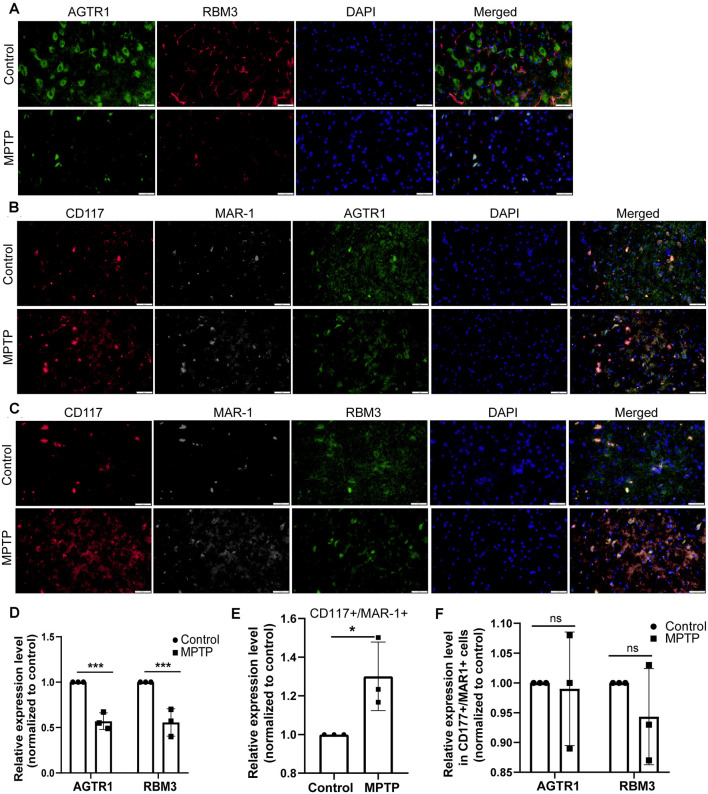
The relative protein expression levels of RBM3 and AGTR1 in the PD animal model. **(A)** The co-localization of AGTR1 and RBM3 in SN of two groups was detected by fluorescence microscope. Scale bars: 50 μm. **(B)** The co-localization of AGTR1, MAR-1, and CD117 in SN of two groups was detected by fluorescence microscope. **(C)** The co-localization of RBM3, MAR-1, and CD117 in SN of two groups was detected by fluorescence microscope. **(D)** The relative expression levels of AGTR1 and RBM3 in SN of the two groups are based on the co-localization of AGTR1 and RBM3. **(E)** The relative co-expression level of CD117/MAR-1 in SN of two groups based on the co-localization of CD117 and MAR-1. **(F)** The relative expression levels of AGTR1 and RBM3 in SN of two groups separately based on the co-localization of AGTR1/CD117/MAR-1 and RBM3/CD117/ MAR-1 (ns *p* > 0.05, *p < 0.05, ***p < 0.001 vs. Control group).

**Figure 12:**
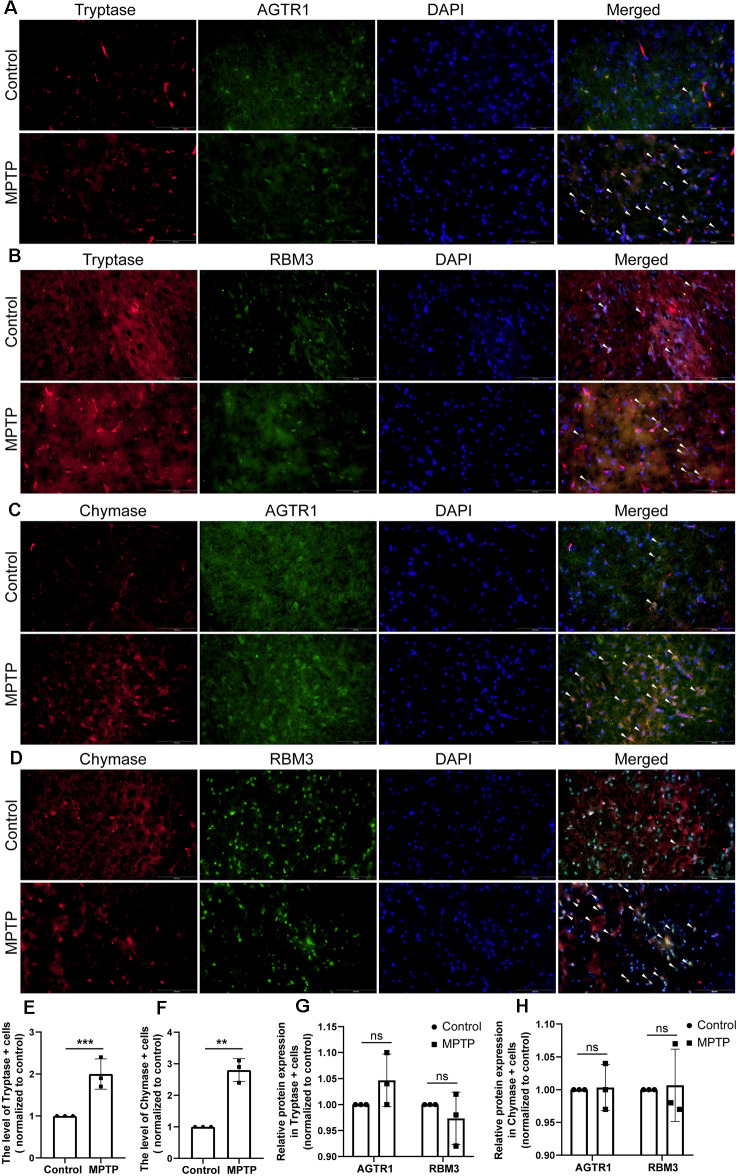
The relative protein expression levels of RBM3 and AGTR1 in the PD animal model. **(A)** The co-localization of AGTR1 and Tryptase in SN of two groups was detected by fluorescence microscope. Scale bars: 100 μm. **(B)** The co-localization of RBM3 and Tryptase in SN of two groups was detected by fluorescence microscope. **(C)** The co-localization of AGTR1 and Chymase in SN of two groups was detected by fluorescence microscope. **(D)** The co-localization of RBM3 and Chymase in SN of two groups was detected by fluorescence microscope. **(E)** The relative expression levels of Tryptase in SN of two groups. **(F)** The relative expression levels of Chymase in SN of two groups. **(G)** The relative expression levels of AGTR1 and RBM3 in SN of two groups separately based on the co-localization of AGTR1/Tryptase and RBM3/ Tryptase. **(H)** The relative expression levels of AGTR1 and RBM3 in SN of two groups separately based on the co-localization of AGTR1/Chymase and RBM3/ Chymase (ns *p* > 0.05, **p < 0.01, ***p < 0.001 vs. Control group).

Moreover, we constructed *in vitro* and *in vivo* PD models to verify the result. The expression levels of RBM3 and AGTR1 in PD cell models based on SH-SY5Y cell line and two PD-related neurotoxins, Rotenone and MPP ^+^, also showed a similar trend, but the trend of RBM3 and AGTR1 was unavailable in SH-SY5Y cell treated with 6-OHDA (Figures 8G–I, Figures 9A–D). Interestingly, compared with the two-cell co-culture system by Transwell, in the co-culture system that was based directly on the co-culture of SH-SY5Y and HMC-1, the expression levels of these two genes in SH-SY5Y and HMC-1 respectively showed more significant differences comparing with the control group (8J–M, Figures 9A–F). After successfully constructing and evaluating MPTP subacute models (10A–E), we analyzed the expression levels of AGTR1 and RBM3 in SN of MPTP mice, which were significantly downregulated compared with the control group (Figures 10F–H, Figures 11A,D). According to co-localization staining, the expression levels of AGTR1 and RBM3 in dopaminergic neurons labeled by tyrosine hydroxylase (TH) of MPTP mice also showed the downregulated trend (Figures 10I,J), but the trend was unavailable in mast cells labeled by CD117, MAR-1, Tryptase, and Chymase simultaneously of MPTP mice (Figures 11B,C,F, Figures 12A–H). Furthermore, CD117/MAR-1, Tryptase, and Chymase staining also verified the increased infiltration of mast cells in MPTP mice (Figures 11B,C,E, 12A–F). Taken together, these results further confirmed that the levels of RBM3 and AGTR1 in neurocytes could be significantly downregulated in both *in vivo* and *in vitro* PD models, and the changes could be shown in neuronal cells directly contacted Should it be “connected” with mast cells.

## Discussion

The arrival of peripheral immune cells at the CNS may have a role in modulating these microglial functions such that subsequent stimuli produce exaggerated responses, and thus affect the outcome in CNS injury and disease (Prinz and Priller, 2017; Greenhalgh et al., 2020; Urban et al., 2020). All lines of evidence provided, it might be speculated that a key trigger to PD pathogenesis is the peripheral immune system that could affect the neuroinflammation of CNS to induce and promote the process of PD (Sim et al., 2020). In this study, using high throughput data and bioinformatic techniques, we provided more robust evidence for the potential roles of infiltrating immunocytes in PD as well as their key molecules, which might be helpful to further illustrate the correlations between the peripheral immune system and PD.

It is generally accepted that T-cell infiltration participated in nigrostriatal dopaminergic neurodegeneration in models of PD (González and Pacheco, 2014). Based on our analysis of the immune infiltration, 10 kinds of immune cells in PDs were identified to be significantly different from those in HCs, which provided new insights into the infiltrating patterns of immunocytes in PD. Mast cell was the most eye-catching immunocyte that had wide correlations with other immune cells and disease states. PD brains showed increased number and activation of mast cells compared with normal brains of non-PD control in both human tissues and animal models (Kempuraj et al., 2019). Our results proved the increased number and activation of mast cells in PD models through the staining of CD117, MAR-1, Chymase, and Tryptase. These proteases from mast cells could activate Protease-activated receptor-2 (PAR-2) that is expressed in neuronal and glial cells and is involved in the development and progression of PD and upregulate neuroinflammation (Liu et al., 2014; Kempuraj et al., 2019; Widera et al., 2019). Additionally, tryptase induces the recruitment and accumulation of mast cells at the site of inflammation through the activation of PAR-2 (Liu et al., 2016). The changes of mast cells also occur together with the activation of microglial and astrocyte. Mast cells can selectively release proinflammatory cytokines/chemokines and neuroactive mediators in pathophysiological conditions (Skaper et al., 2014; Hendriksen et al., 2017; Kempuraj et al., 2018). These cytokines not only could active microglial and astrocytes through matched receptors, but also break down BBB and attract peripheral immune cells to increase inflammatory infiltration (Hendriksen et al., 2017). The glial cells can also interact with mast cells and accelerate neuroinflammation, and the crosstalk may act as a novel therapeutic target for PD.

RBM3 is a neuroprotective cold-shock protein and can improve neurological function. According to previous researches, knockdown of RBM3 can aggravate apoptosis inducted by rotenone and 1-methyl-4-phenylpyridinium (MPP+), while overexpression of RBM3 not only reduces this apoptosis, but also restores structural synaptic plasticity, and mediates neuroprotection against rotenone by inhibiting the MAPK signaling of p38, JNK, and ERK (Peretti et al., 2015; Yang et al., 2018, 2019). In a word, the relationship between RBM3 and PD is worthy of further investigation.

The angiotensin II type 1 receptor (AT1R) is coded by AGTR1 and is termed as the component of the renin-angiotensin system. Compared with the matched controls, radiolabeled AT1R recognition site levels were significantly decreased by approximately 70%, 70%, and 90% in the caudate nucleus, putamen, and SN (Ge and Barnes, 1996); and total cellular AT1R expression in SN DA neurons is reduced with disease progression (Zawada et al., 2015). However, AT1R upregulation can lead to the release of pro-inflammatory cytokines and subsequent inflammation to induce dopaminergic cell death and dysfunction, and its antagonist could reduce dopaminergic neuron degeneration introduced by a neurotoxin in SN (Grammatopoulos et al., 2007; Sathiya et al., 2013). The results of model-based studies seem to be contrary to the clinical phenomenon, but it also indicates that the role of AGTR1 in PD is complex and needs further exploration.

Our study had several limitations that should be acknowledged. First, the method combined with metagenes and ssGSEA could not accurately identify immune cell subtypes from bulk RNA-Seq data, since some of these metagenes are controversial to represent specific immune cell subpopulations. Although, we analyzed the expression of RBM3 and AGTR1 in dopaminergic neurons and mast cells in PD models using co-localization staining, the relationship between other cell types and these two genes remains unclear. It is necessary to use single-cell RNA-Seq or other methods for verifying our results and illustrating the roles of RBM3 and AGTR1 in different cell types of PD. Second, we did not illustrate the correlations between immune infiltration and clinical symptoms due to the lack of complete clinical information in the included datasets. Then, although we collected multiple datasets in the public database, the number of samples in the present study was not large enough, which required further real-world studies to verify the current results. Besides these, the current results also should be validated in human PD brain specimens. Although mast cell plays key roles in PD, the source of mast cell is unknown and need to be studied. Finally, although our findings were verified in *in vitro* and *in vivo* PD models and other published researches, functional experiments based on models that could better imitate the real state of PD, like induced pluripotent stem cells from PD patients and transgenic animal models are still necessary to reveal the roles of RBM3 and AGTR1 played in PD.

In conclusion, based on the gene expression profiling, the immune landscape of PDs was preliminarily constructed. This study revealed core immunocytes and molecules significantly associated with peripheral immune cell infiltration in SN of PD patients. Our findings will help enhance our cognition of immune cell infiltration heterogeneity and complexity, as well as provide promising targets for immunotherapy in patients with PD.

## Data Availability Statement

The original contributions presented in the study are included in the article/Supplementary Material, further inquiries can be directed to the corresponding authors.

## Author Contributions

XZ, ZS, YL, LJ, and SL designed the study. XZ, SX, QL, and CL performed the data analysis. XZ, ZS, and SX wrote the manuscript. YL, LJ, and SL reviewed and edited the manuscript. All authors contributed to the article and approved the submitted version.

## Conflict of Interest

The authors declare that the research was conducted in the absence of any commercial or financial relationships that could be construed as a potential conflict of interest.

## Footnotes

^1^http://www.ncbi.nlm.nih.gov/geo
